# Atomistic Simulation of Lysozyme in Solutions Crowded by Tetraethylene Glycol: Force Field Dependence

**DOI:** 10.3390/molecules27072110

**Published:** 2022-03-25

**Authors:** Donglin Liu, Yejie Qiu, Qing Li, Haiyang Zhang

**Affiliations:** Department of Biological Science and Engineering, School of Chemistry and Biological Engineering, University of Science and Technology Beijing, Beijing 100083, China; donglinliuu@sina.cn (D.L.); yejieqiu@sina.cn (Y.Q.); qingli_ustb@sina.com (Q.L.)

**Keywords:** molecular crowding, molecular dynamics simulation, tetraethylene glycol, protein diffusion

## Abstract

The behavior of biomolecules in crowded environments remains largely unknown due to the accuracy of simulation models and the limited experimental data for comparison. Here we chose a small crowder of tetraethylene glycol (PEG-4) to investigate the self-crowding of PEG-4 solutions and molecular crowding effects on the structure and diffusion of lysozyme at varied concentrations from dilute water to pure PEG-4 liquid. Two Amber-like force fields of Amber14SB and a99SB-*disp* were examined with TIP3P (fast diffusivity and low viscosity) and a99SB-*disp* (slow diffusivity and high viscosity) water models, respectively. Compared to the Amber14SB protein simulations, the a99SB-*disp* model yields more coordinated water and less PEG-4 molecules, less intramolecular hydrogen bonds (HBs), more protein–water HBs, and less protein–PEG HBs as well as stronger interactions and more hydrophilic and less hydrophobic contacts with solvent molecules. The a99SB-*disp* model offers comparable protein–solvent interactions in concentrated PEG-4 solutions to that in pure water. The PEG-4 crowding leads to a slow-down in the diffusivity of water, PEG-4, and protein, and the decline in the diffusion from atomistic simulations is close to or faster than the hard sphere model that neglects attractive interactions. Despite these differences, the overall structure of lysozyme appears to be maintained well at different PEG-4 concentrations for both force fields, except a slightly large deviation at 370 K at low concentrations with the a99SB-*disp* model. This is mainly attributed to the strong intramolecular interactions of the protein in the Amber14SB force field and to the large viscosity of the a99SB-*disp* water model. The results indicate that the protein force fields and the viscosity of crowder solutions affect the simulation of biomolecules under crowding conditions.

## 1. Introduction

Biomolecules perform a variety of functions in living cells where they are crowded by many macromolecules such as proteins, nucleic acids, and polysaccharides. The concentration of macromolecular crowders goes up to 400 g/L, and these crowders occupy 5–40% of the total volume physically [1]. The resulting excluded volume affects the stability and folding of proteins [2,3,4,5] as well as enzyme activity [6,7,8] via a reduced diffusion and collision [9] between the molecules. Besides such “hard” interactions (steric, entropic effects), “soft” interactions (transient, enthalpic effects) contribute significantly to the structural, dynamic, and thermodynamic properties of biomolecules in crowded environments as well [10,11,12]. Crowding and confinement of biomolecules in concentrated solutions have attracted considerable attention in recent years for unveiling the underlying behaviors and mechanisms via both experimental and computational approaches [7,13,14,15,16,17,18,19,20,21,22,23].

A number of factors such as shape, size, concentration, and composition of molecular crowders were reported to be influential [18,24,25]. Polyethylene glycol (PEG) was often used as a crowder to mimic the cell-like environment [6,26,27,28,29,30]. For instance, Nolan and coworkers reported a noticeable increase in the thermal stability and catalytic activity of *β*-galactosidase in the presentence of PEG with a concentration of 25% and 35% (*w/v*) [29]. Wang and coworkers indicated that the increase in the residual activity of lysozyme was ascribed to the fact that the addition of PEG suppressed enzyme aggregation via a strong PEG–protein interaction and stabilized the protein secondary structure somewhat [6]. On the contrary, soft interactions with PEG were also observed to induce structural changes and destabilize proteins [28,31,32,33,34]. For the nucleic acids, low molecular weight PEGs were observed to decrease their thermodynamic stability, while high molecular weight PEGs showed the opposite [35]. PEG-induced destabilization of biomolecules is likely due to the stronger “soft” interactions relative to the “hard” interactions of excluded volume; the former offers either favorable or unfavorable contributions, while the latter leads to compacted structures and is usually thermodynamically favorable [12].

Reduced available volume in crowed environments has a consequence of slow diffusion for biomolecules due to the more contacts between molecules and increased viscosity compared to a diluted solution [20,36,37,38,39,40,41]. The anomalous diffusion may help the biomolecules find a nearby target [42] or increase the contact time of binding partners by preventing the escape of the enzymes to the bulk solution [43]. Munishkina and coworkers demonstrated that the excluded volume effects in a low concentration of PEG crowders accelerated protein fibrillation, while a high concentration slowed down the fibrillation, likely due to the increased viscosity affecting the protein diffusion [44]. Despite a high solution viscosity, the crowders are likely not distributed evenly in the system, and the non-uniform crowding may produce an enhanced transport of the molecules in crowder gradients [45].

Computational approaches are a valuable supplement to experimental measurements for the investigation of the structure, dynamic, association, and charge regulation [14,20,23,40,46,47,48,49,50,51] as well as the liquid-liquid phase separation of biomolecules in a crowded environment [52,53,54,55,56,57]. Despite the expensive computational cost in modeling crowded environments, model accuracy is also an issue. Modern force field models of, for instance, proteins, were argued to predict too compacted (stable) structures [58,59,60] and artificial protein aggregation [20,61,62] due to stronger protein–protein interactions relative to protein–water interactions. A popular solution is to strengthen the interactions between protein and water via choosing new water models with a large dispersion coefficient (such as TIP4P/D [63], OPC [64,65], and a99SB-*disp* [66]) or scaling the interactions directly without modifying water–water interactions (such as the Amber99SBws [60] with TIP4P/2005 [67] water model and the CHARMM36m [68] with TIP3P [69]). Note that a new water model usually has different water–water interactions, which likely affects the balance of protein–water interactions as well [70].

The choice of force field models for proteins and crowders is of vital importance for a reliable explanation and prediction of molecular crowding in highly concentrated solutions, in particular, for the association and diffusion properties of proteins. The impact of crowding is a combination of excluded volume effects and non-specific interactions, both of which are complex and may differ from case to case [71]. A number of reports focused on the macromolecular crowding effects of relatively large molecular weight crowders. Less attention was paid to the effects of low molecular weight crowders (referred to as molecular crowding), although theoretical and experimental observations provided evidence that “smaller molecules crowd better” for some cases [72,73]. For instance, the crowders with a medium size showed very similar crowding effects to that with larger macromolecules such as proteins and RNA [72].

Here we chose tetraethylene glycol (PEG-4) as a small molecular crowder and simulated a model protein lysozyme in PEG-4 solutions with a wide range of concentrations. Two Amber-like protein force fields of Amber14SB [74] and a99SB-*disp* [66] were examined. The former is in conjunction with the popular TIP3P water model [69], having a fast diffusivity and low viscosity; the latter is with the a99SB-*disp* water model [66], having a slow diffusivity and high viscosity [75]. The a99SB-*disp* force field was designed for both folded and disordered protein states [66], while the performance of Amber14SB appeared to be case-dependent [70]. Effects of crowed conditions on the structure and translational diffusion of lysozyme were addressed in detail as well as the comparison between both force fields. This work highlights a force field dependence in the modeling of crowding environments and is useful for a better understanding of the crowding effects from computational simulations.

## 2. Results and Discussion

### 2.1. Density and Viscosity of PEG-4 Solutions

**Figure 1:**
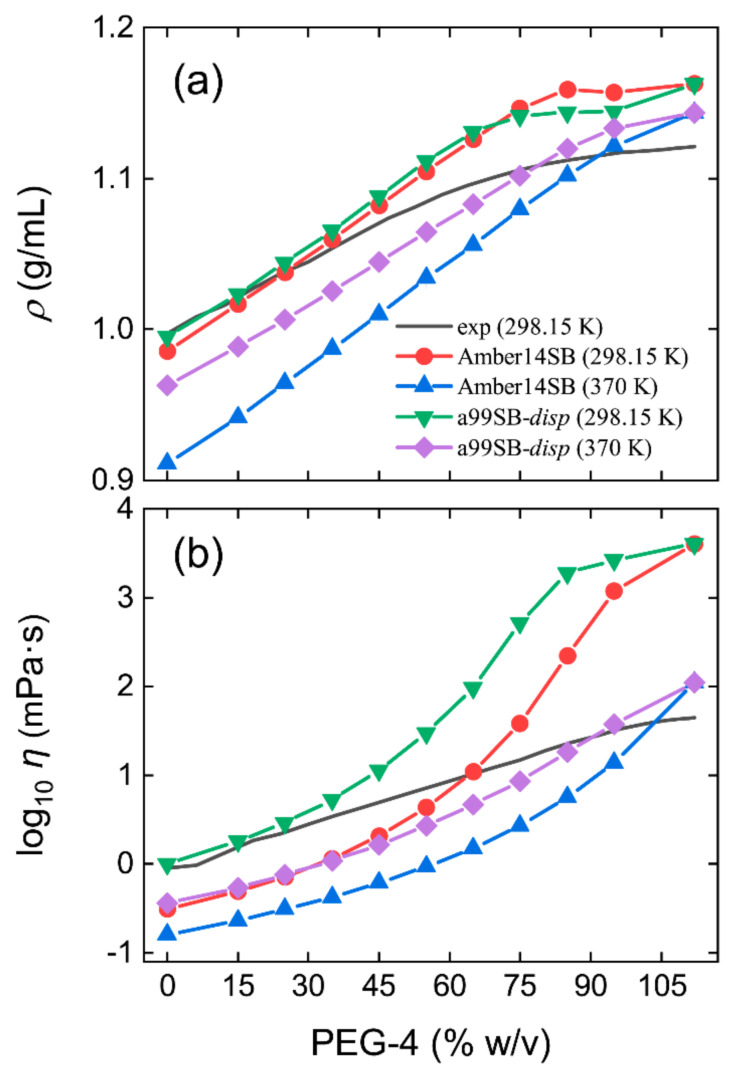
Simulated density (*ρ*, **a**) and viscosity (*η*, **b**) of PEG-4 solutions as a function of concentrations at 298.15 and 370 K using Amber14SB and a99SB-*disp* force fields. Black solid lines are experimental observations [78].

The Amber-like force fields of Amber14SB [74] and a99SB-*disp* [66] examined in this work were developed for use with TIP3P [69] and a99SB-*disp* [66] water models, respectively. The model of PEG-4 was identical in our simulations of PEG-4 solutions using both force fields. The simulated densities (*ρ*) of pure water at 298.15 K were 0.9857 and 0.9952 g/mL for TIP3P and a99SB-*disp* water models (Figure 1a), respectively, in good agreement with the experimental measurement of 0.9971 g/mL [76]. TIP3P gave a prediction of 0.9111 g/mL at 370 K, an underestimation by 5% compared to the experiment of 0.9606 g/mL [76]. The a99SB-*disp* model reproduced the water density at a high temperature of 370 K accurately with an estimation of 0.9630 g/mL (Figure 1a). Using both water models, the density of PEG-4 solutions at 298.15 K could be reproduced accurately when the concentration was smaller than 35% *w/v* (Figure 1a). At higher concentrations, a larger density than the experiments was observed, and the density of pure PEG-4 liquid was overestimated by 4%. Both water models displayed similar density profiles of PEG-4 solutions at 298.15 K, which increased, as expected, with the increasing PEG-4 concentrations. For a high temperature of 370 K, however, TIP3P led to a smaller density of the PEG-4 solutions than that of a99SB-*disp* (Figure 1a), probably due to the underestimated water density at 370 K with the TIP3P model.

The TIP3P and a99SB-*disp* models estimated the shear viscosity of pure water at 298.15 K to be 0.31 and 1.01 mPa·s (Table S1 in the Supporting Information). Compared to the experiment of 0.89 mPa·s [77], TIP3P displayed a too low viscosity with an underestimation by 65%, a well-known drawback of the model, while the a99SB-*disp* model gave a better prediction with an overestimation by 13%. The simulated viscosities were 0.16 and 0.36 mPa·s at 370 K and differed from the experiment of 0.28 mPa·s [77], with an underestimation by 43% for TIP3P and an overestimation by 29% for a99SB-*disp*. We noted a slow convergence of viscosity calculations in PEG-4 solutions. For a low concentration like pure water, a length of within 1 ns gave a reasonable estimation, and 10 ns appeared necessary for a better statistic (Figure S1 in the SM). For the pure PEG-4 solution, we needed at least 30 ns to ensure convergence (Figure S1).

The use of the TIP3P water model largely underestimated the viscosity of PEG-4 solutions for concentrations of smaller than 55% *w/v*, and the a99SB-*disp* model slightly overestimated the viscosities at a low concentration of smaller than 35% *w/v* (Figure 1b and Table S1). For highly concentrated PEG-4 solutions, both water models dramatically overestimated the viscosities of PEG-4 solutions. The viscosity of a pure PEG-4 liquid was estimated to be 4057.45 ± 279.95 mPa·s, two orders of magnitude larger than the experiment of 44.63 mPa·s at 298.15 K [78]. As the temperature increased, as expected, the density and viscosity of PEG-4 solutions decreased (Figure 1). It appeared that the temperature had a large influence on the simulated viscosity, and the viscosity of the pure PEG-4 liquid was reduced to 109.42 ± 1.05 mPa·s at 370 K (Table S1). Note that the viscosity results in Figure 1b were plotted on a log10 scale; on a linear scale, the true magnitude of the disagreement would be more apparent (Table S1).

### 2.2. Self-Crowding of PEG-4

Diffusion constants of water and PEG-4 in different PEG-4 concentrations were calculated to investigate the self-crowding effects on the solvent diffusivity, as given in Tables S2 and S3, respectively. For the pure water (0% *w/v*), TIP3P and a99SB-*disp* models gave diffusion constants of 6.13 ± 0.10 and 1.91 ± 0.08 in units of 10^−5^ cm^2^/s at 298.15 K, and a faster diffusivity was observed at 370 K with a prediction of 13.80 ± 0.31 and 6.37 ± 0.15 × 10^−5^ cm^2^/s, respectively (Table S2). Compared to the experiment of 2.30 × 10^−5^ cm^2^/s at 298.15 K [79], TIP3P showed a large overestimation by 167%, while an underestimation by 17% was detected for a99SB-*disp*. This finding was attributed to the low viscosity of TIP3P and the high viscosity of a99SB-*disp* (Figure 1b and Table S1).

**Figure 2:**
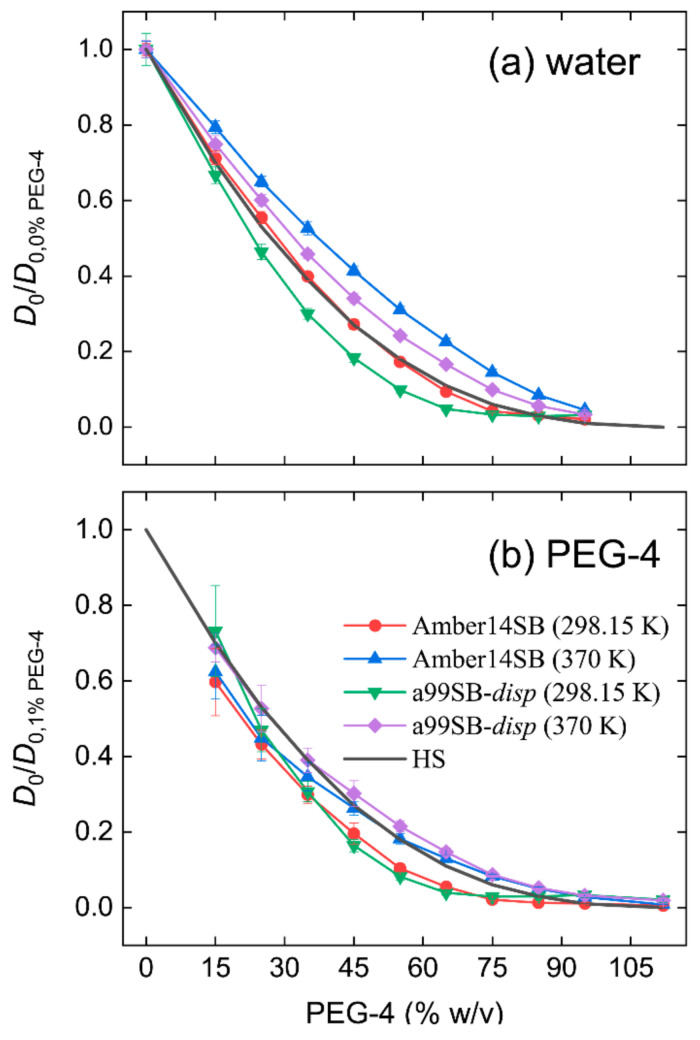
Diffusion constants of water (**a**) and PEG-4 (**b**) in different concentrations of PEG-4 solutions. The calculated diffusion constants are normalized to be one in the cases of 0% and 1% *w/v* for water and PEG-4, respectively. A decrease in the diffusion constants for hard spheres based on the Enskog theory (Equation (1)) is given in black for comparison.

As the PEG-4 concentration increased, the diffusion constants of water molecules decreased due to the crowding of PEG-4 molecules (Figure 2 and Table S2). After normalizing the calculated diffusion constants to be one for the case of 0% *w/v* (i.e., pure water), we found that the drop in water diffusivity was faster at 298.15 K than at 370 K, and the solutions with the a99SB-*disp* water model displayed a faster drop than that with the TIP3P model (Figure 2a). This was ascribed to the fact that the relatively small viscosity at a high temperature was favorable for particle diffusion and that the TIP3P-related systems had a smaller viscosity than that with the a99SB-*disp* model.

The Enskog theory predicts a decline in the diffusion constants (*D*_HS_) for hard spheres via the volume fraction (∅) of the crowders (Equation (1)) [80,81].

(1) $$\frac{D_{\mathrm{HS}}}{D_{\mathrm{HS}0}}=\frac{{\left(1-\emptyset \right)}^{3}}{1-0.5\emptyset }$$

where *D*_HS0_ is the diffusion constant of the hard spheres in pure solvent. The calculated volume fractions of the PEG-4 molecules were given in Table S3. The decrease for water molecules in PEG-4 solutions with the TIP3P water model at 298.15 K was in good agreement with the Enskog theory, as indicated by the comparison of red and black lines in Figure 2a.

Similar to water molecules, diffusion constants of PEG-4 decreased with the increasing concentration of PEG-4 solutions and with the decreasing temperature (Table S4). We normalized the PEG-4 diffusivity to be one for the 1% *w/v* system, which represented the diffusion of PEG-4 in dilute aqueous solution. Although the used water models and the system viscosities were different, the drops in the normalized diffusion constants of PEG-4 displayed similar profiles for both force fields (Figure 2b). This was likely due to the very similar interactions between PEG-4 molecules because the force used identical PEG-4 parameters. The drop profiles at 370 K coincided with the prediction for the hard spheres, while the cases at 298.15 K showed a slight underestimation (Figure 2b).

### 2.3. Structure and Thermodynamics of Lysozyme

#### 2.3.1. Overall Stability

**Figure 3:**
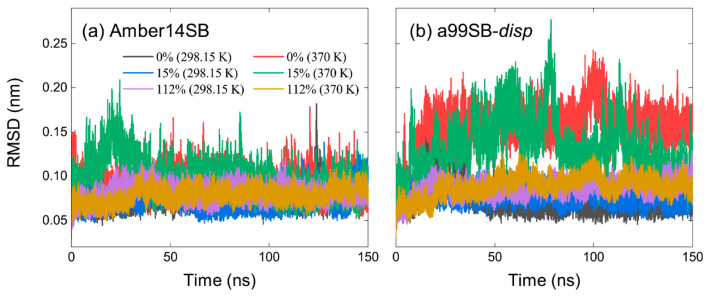
Root-mean-square deviation (RMSD) of lysozyme backbone from crystal structure as a function of simulation time for Amber14SB (**a**) and a99SB-*disp* (**b**) force fields with PEG-4 concentrations of 0%, 15%, and 112% (*w/v*) at 298.15 and 370 K.

**Figure 4:**
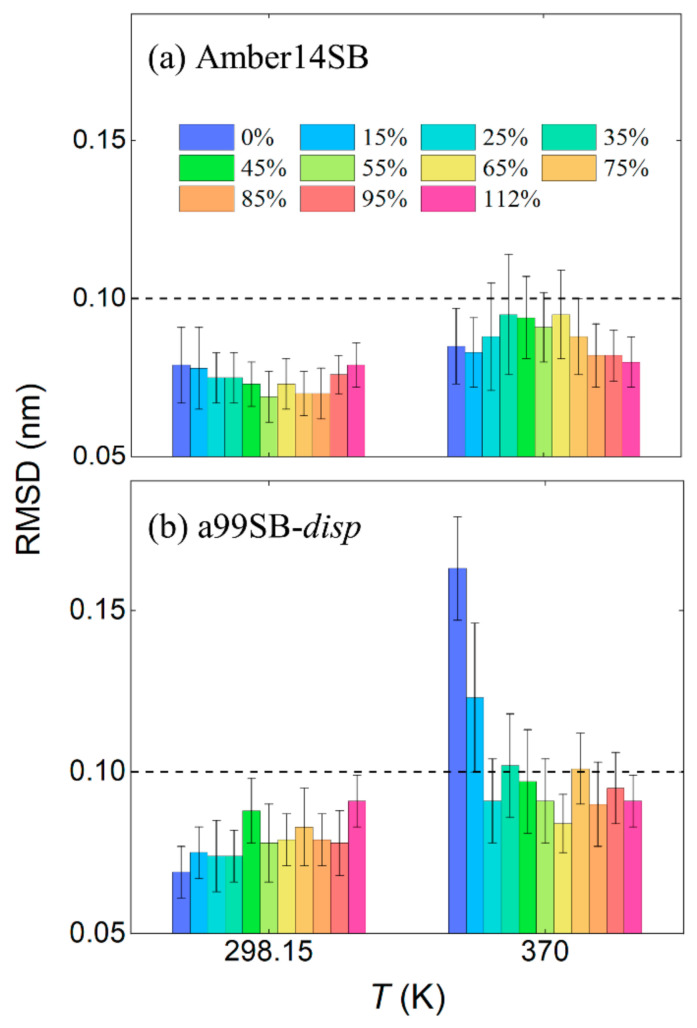
Root-mean-square deviation (RMSD) of lysozyme backbone from crystal structure in different PEG-4 concentrations (*w/v*) at 298.15 and 370 K for Amber14SB (**a**) and a99SB-*disp* (**b**) force fields. The dashed lines indicates a value of RMSD = 0.1 nm.

The root-mean-square deviations (RMSDs) of the lysozyme backbone from the crystal structure reached equilibrium after 100 ns simulations, as indicated by the protein systems with the concentrations of 0%, 15%, and 112% *w/v* in Figure 3a,b for the Amber14SB and a99SB-*disp* force fields, respectively. The last 50 ns of MD trajectories were used for data collection. No significant differences were observed for the equilibrated RMSDs in different PEG-4 concentrations at 298.15 and 370 K using the Amber14SB force field, although the values at 370 K appeared slightly larger than that at 298.15 K (Figure 4a). The same went for the simulations with the a99SB-*disp* force field at 298.15 K (Figure 3b and Figure 4b). Increasing the temperature to 370 K, however, the protein lysozyme showed a large RMSD of >0.1 nm at low concentrations of 0% and 15% *w/v*. In high concentrations of PEG-4 solutions at 370 K, the RMSDs were maintained within ~0.1 nm, indicating that the PEG-4 crowding stabilized the overall structure of lysozyme to some extent.

**Figure 5:**
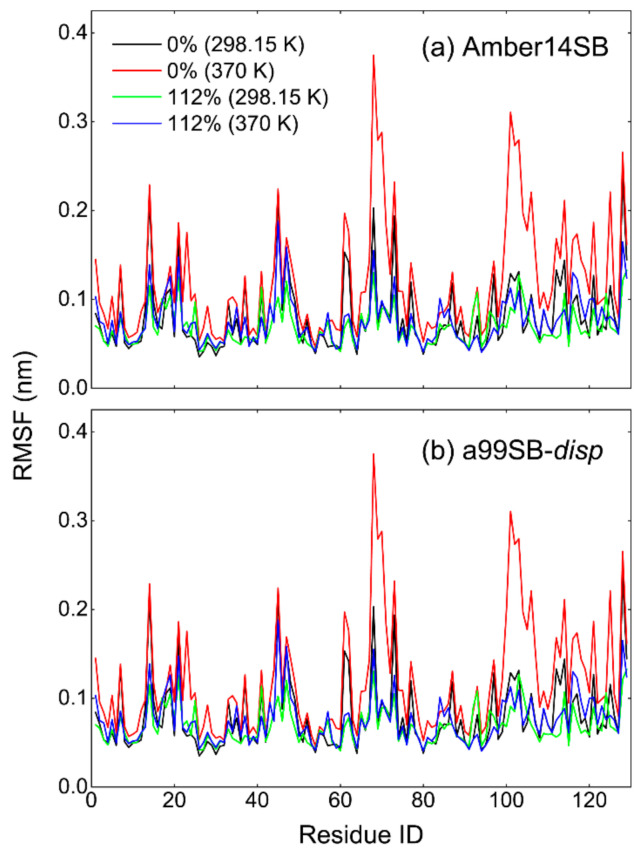
Root-mean-square fluctuation (RMSF) of lysozyme residues for Amber14SB (**a**) and a99SB-*disp* (**b**) force fields with PEG-4 concentrations of 0%, 15%, and 112% (*w/v*) at 298.15 and 370 K.

Despite the similarity of average RMSDs, the RMSD distributions appeared to display a narrower landscape for high PEG-4 concentrations of >75% *w/v* than that in low concentrations, in particular for the simulations at 370 K (Figure S2). This means that the flexibility of the protein structure was restricted somewhat, probably due to the high viscosity of protein/PEG-4 solutions (Table S5). The restricted flexibility can be reflected by a small root-mean-square fluctuation (RMSF) of lysozyme residues as well (Figure 5). Although the system viscosities for the low concentrations at 370 K (Table S5) were significantly reduced compared to that at 298.15 K, the Amber14SB force field yielded a relatively smaller RMSD of the protein backbone than the a99SB-*disp* force field. These discrepancies likely arose from the difference in the protein force field; the a99SB-*disp* model was designed for both folded and disordered proteins, while the Amer14SB model was mainly for the folded proteins. This means that a protein modeled by a99SB-*disp* was likely less stable than that by Amber14SB. The simulated temperature of 370 K was very close to the thermal denaturation midpoint (360 K) of lysozyme [2,82]; however, we did not observe considerable changes in the protein structure. This was likely due to the used force field models producing too strong intramolecular interactions of the protein and/or too large viscosity of the crowded solutions.

**Figure 6:**
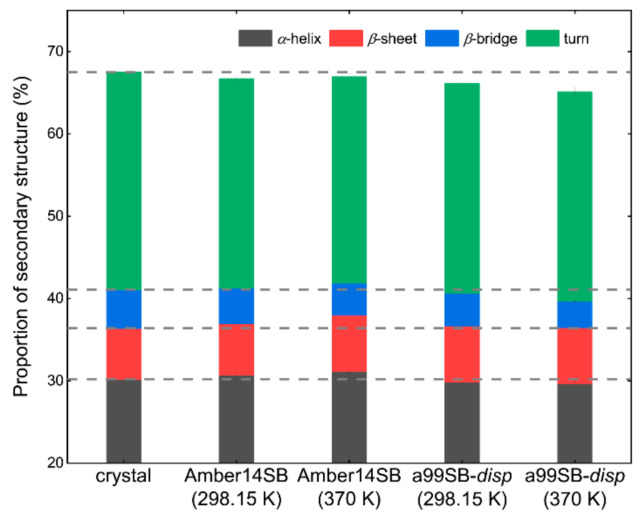
Proportion of protein secondary structures for the simulation of lysozyme in pure water (0% *w/v*) using Amber14SB and a99SB-*disp* force fields at 298.15 and 370 K. Dashed gray lines indicate the values of the crystal structure.

The total proportion of secondary structures of simulated lysozyme at 298.15 K and 370 K in water using the Amber14SB force field was similar to that in the crystal structure (Figure 6), although the simulations tended to yield a slightly larger fraction of *α*-helix and *β*-sheet and a slightly smaller fraction of *β*-bridge and turn (Table S6). The a99SB-*disp* force field produced a smaller proportion of the protein secondary structures than that of Amber14SB (Figure 6), due to the slight underestimation of *α*-helix, *β*-bridge, and turn structures (Table S6). Note that both force fields predicted a slightly larger fraction of *β*-sheet structure. Because the formation of *β*-sheet was argued to increase the possibility of protein aggregation [6,83,84], both force fields should be used with caution in simulations of concentrated systems crowded by proteins. Note that no significant changes were observed in the protein secondary structures for all of the simulated systems (Table S6). Increasing the temperature to, for instance, 500 K, Wang and coworkers observed dramatic changes in the lysozyme conformation [6]. The experimental melting point of lysozyme was roughly 360 K [2,82], and we did not try to simulate the protein at a high temperature over 370 K because the unfolded protein did not allow a meaningful and direct comparison of protein diffusion in different crowed environments (discussed in the following; Section 2.4).

#### 2.3.2. Solvent Distribution

**Figure 7:**
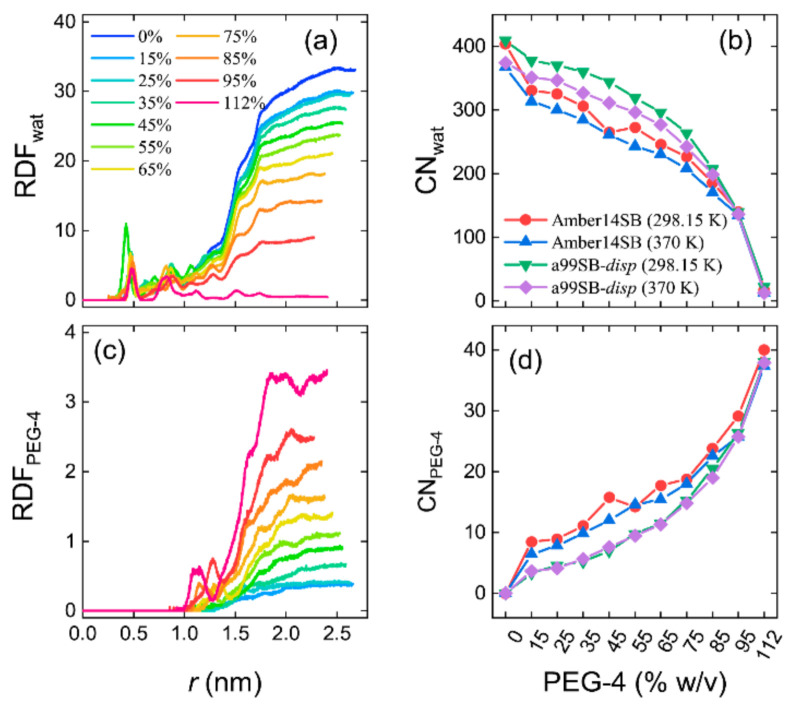
Non-normalized radial distribution functions (RDFs) and the coordinated number (CN) of solvent molecules within the hydrodynamic radius (1.87 nm) of lysozyme for water (**a**,**b**) and PEG-4 (**c**,**d**) for the simulations of lysozyme in different PEG-4 concentrations using Amber14SB and a99SB-*disp* force fields at *T* = 298.15 and 370 K. Panels (**a**,**c**) are the RDFs with the a99SB-*disp* force field, and similar landscapes were observed for the Amber14SB force field.

We used non-normalized radial distribution functions (RDFs) to characterize the number density of solvent molecules distributed around the protein lysozyme. In pure water (0% *w/v*), the RDF leveled off at a large protein–solvent distance (*r*) with a number density of 33.3 nm^−3^, equal to the number density of a pure water liquid (Figure 7a). The radius of gyrate of lysozyme for the crystal structure was 1.4 nm. Within this radius, two obvious peaks were observed at *r* = 0.45 and 0.88 nm, corresponding to the water molecules in the interior of the protein. From the cumulative RDFs, we obtained the coordination number (CN) of water molecules with the hydrodynamic radius (1.87 nm) of the protein, as shown in Figure 7b. We can see that the simulations with the a99SB-*disp* force field produced more coordinated water molecules (CN_water_) around lysozyme than that with the Amber14SB force field. As expected, the CN_water_ decreased with the increasing PEG-4 concentrations (Figure 7b), as verified by the decreased number density of water molecules at *r* > 1.87 nm (Figure 7a).

RDF profiles of PEG-4 around protein showed that PEG-4 was not able to enter the interior of the lysozyme and, in high concentrations, PEG-4 approached the protein surface, as indicated by a peak at *r* = 1.2–1.3 nm (Figure 7c). Das and Sen indicated that a relatively small crowder (dextran, 6 kD) was likely to penetrate the interior of human serum albumin, facilitating a destabilizing soft interaction between the crowder and protein, whereas this might not happen for larger crowders (dextran, 40 kD and 70 kD) [85]. PEG-4 is quite small, as compared to the commonly used macromolecular crowders, and therefore, such penetration appeared to depend on the compactness of the protein and the size of the crowders. The coordination number of PEG-4 within 1.87 nm of protein increased with the increasing PEG-4 concentrations, and unlike CN_water_, the a99SB-*disp* force field supported fewer interacting PEG-4 molecules with the protein than that with the Amber14SB (Figure 7d). Note that the number of solvent molecules (water + PEG-4) was identical for the simulations with both force fields. The discrepancy in the CN of solvent molecules indicated a difference in the modeling of protein–solvent interactions for the used protein force fields. For all of the systems, increasing temperature led to a decrease in the CN of water molecules, whereas the temperature had little influence on the CN of PEG-4 molecules (Figure 7).

#### 2.3.3. Hydrogen Bonds

**Figure 8:**
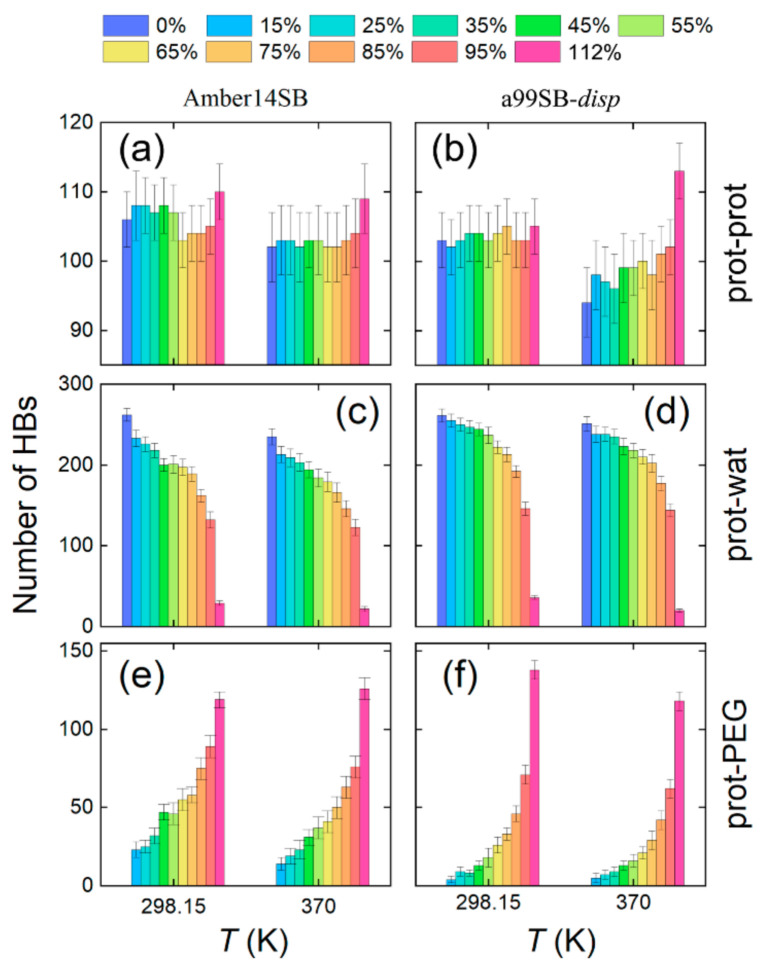
Number of intramolecular hydrogen bonds (HBs) for protein (prot-prot, **a**,**b**) and HBs between protein and water (prot-wat, **c**,**d**) and between protein and PEG-4 (prot-PEG, **e**,**f**) for the simulations of lysozyme in different PEG-4 concentrations using Amber14SB (**left**) and a99SB-*disp* (**right**) force fields at 298.15 and 370 K.

Intramolecular hydrogen bonds (HBs) of proteins, denoted as prot-prot HBs here, are of importance to maintain structural stability. For the simulations with the Amber14SB force field, we detected 106 ± 2 prot-prot HBs at 298.15 K; increasing the temperature to 370 K interrupted the HBs slightly with an HB number of 103 ± 1 (Figure 8a). The PEG-4 crowding appeared to have no obvious influence on the prot-prot HBs when PEG-4 concentrations were smaller than 95% *w/v* (that is, in the presence of enough water molecules). For 112% *w/v*, only 70 water molecules were present, and a larger number of prot-prot HBs was detected compared to the case of 0% *w/v* (Figure 8a). Similar findings were observed for the a99SB-*disp* force field at 298.15 K (Figure 8b). For a high temperature of 370 K, however, the prot-prot HBs were largely interrupted, and the HB number seemed to increase with the increasing PEG-4 concentration (Figure 8b).

As the PEG-4 concentration increased, the number of water molecules decreased, and the number of PEG-4 molecules increased. As a result, the HBs between lysozyme and water (prot-wat) decreased, and the HBs between lysozyme and PEG-4 (prot-PEG) increased. This is evidence of soft interactions taking over at high PEG-4 concentrations, which are often enthalpically driven and can stabilize or destabilize the proteins [12]. Compared to the Amber14SB force field, the prot-wat HBs dropped at a slower pace for the a99SB-*disp* force field (Figure 8, panels c and d), and the prot-PEG HBs grew at a faster pace (Figure 8, panels e and f). Interestingly, the a99SB-*disp* force field supports more prot-wat HBs than Amber14SB, while the case of the prot-wat HBs showed the opposite. These findings are in line with the solvent distributions around the protein (Figure 7) and can be explained by the fact that the a99SB-*disp* force field was designed to strengthen protein–water interactions using the a99SB-*disp* water model with a large dispersion coefficient. As a result, the solute–solute interactions appeared to be weakened somewhat in an indirect way.

#### 2.3.4. Interaction Energies

**Figure 9:**
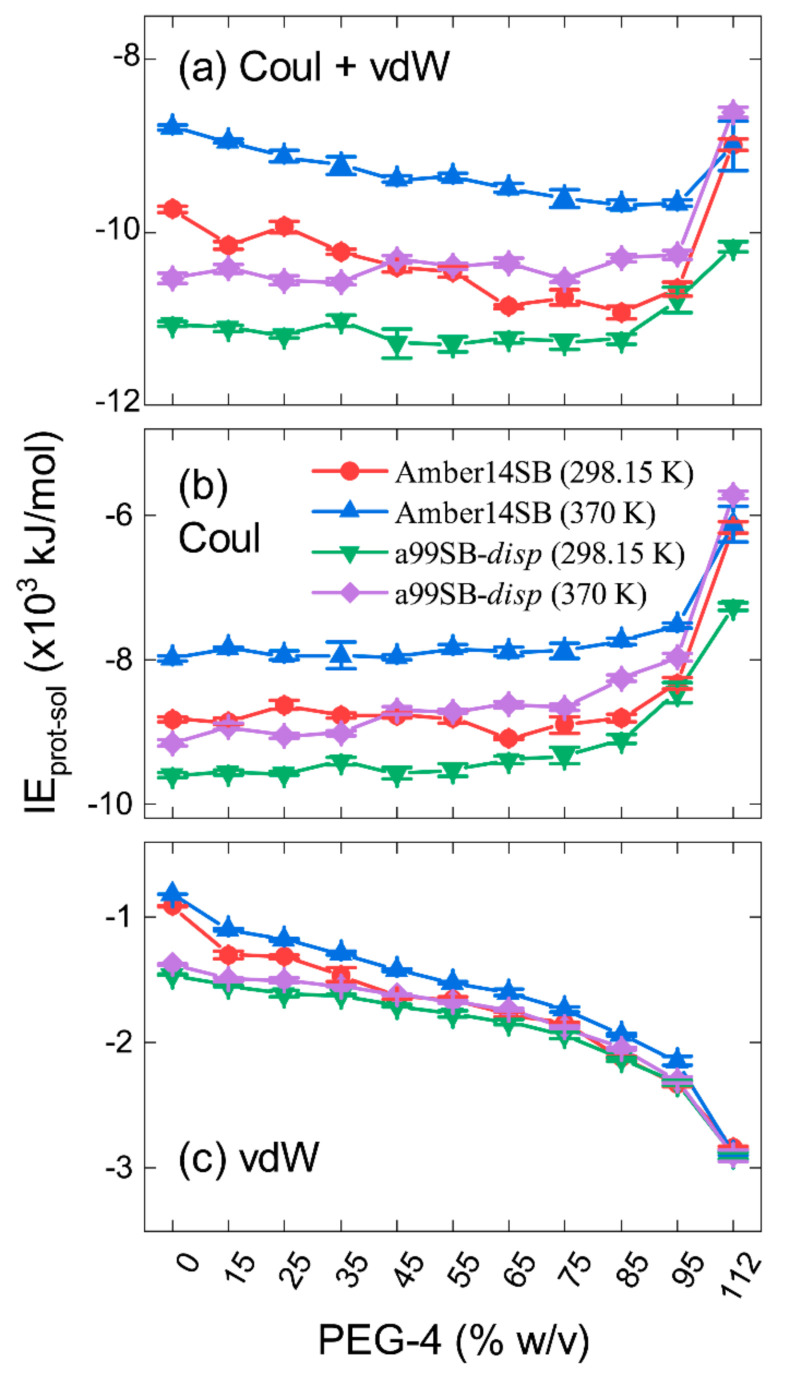
Nonbonded interaction energies (IE, **a**) between lysozyme and solvent molecules (water + PEG-4) for the simulations of lysozyme in different PEG-4 concentrations using Amber14SB and a99SB-*disp* force fields at 298.15 K and 370 K. Contributions from Coulomb (Coul) and vdW interactions to IE are given in panels (**b**,**c**), respectively. Individual contributions from water and PEG-4 are given in Figure S3.

When the PEG-4 concentration was smaller than 95% *w/v*, nonbonded interaction energies (IE) between the protein and solvent molecules (including water and PEG-4) decreased with the increasing concentration for the Amber14SB force field, whereas no obvious changes in the IE were observed for the a99SB-*disp* force field (Figure 9a). There were no significant changes in the Coulomb contributions (IE_Coul_) from solvent molecules (Figure 9b), and the drop in the IE for the Amber14SB was mainly due to the decrease in the van der Waals (vdW) interactions (IE_vdW_) between the protein and solvent molecules (Figure 9c). A slight increase in IE_Coul_ and a slight decrease in IE_vdW_ were observed for the a99SB-*disp*, keeping the total IE almost unchanged (Figure 9). The Amber14SB appeared to give weaker protein–solvent interactions than the a99SB-*disp* due to the weaker interactions of both Coulomb and vdW parts. For all of the cases, a higher temperature corresponded to a weaker interaction.

The decomposition of IE into individual contributions from water and PEG-4 showed that the a99SB-*disp* force field tends to produce stronger prot-wat and weaker prot-PEG interactions than the Amber14SB (Figure S3); this is in line with the solvent distributions in Figure 7 and the HB numbers in Figure 8. We can see that the PEG-4 crowding allowed the crowder to have more of a chance of interacting with PEG-4, as indicated in Figure S3 by the increase in prot-wat interaction energies (less negative) and the decrease in prot-PEG interactions (less negative). Even so, it was possible that the replacement of water molecules by PEG-4 maintained the protein–solvent interactions in a steady state, independent of the crowder concentration, as it did with the a99SB-*disp* force field in Figure 9a. In such a case, the “hard” interactions (excluded volume/steric effects) played a decisive role in the behaviors of biomolecules in the crowding environments. When only a small amount of water existed, like the case of 112% *w/v*, the increment in the IE_vdW_ of prot-PEG interactions was smaller than the decrement in the IE_Coul_ of prot-wat interactions (Figure S3), leading to a weaker interaction between protein and solvent than that in pure water (Figure 9a). The weak protein–solvent interactions likely endowed the protein with structural rigidity and stability, as observed in the concentration of 112% *w/v* (Figure 3, Figure 4 and Figure 5).

The Amber14SB force field yielded stronger intramolecular interactions of the protein than the a99SB-*disp*, and a high temperature interrupted the intramolecular interactions to some extent (Figure S4). This indicated that the Amber14SB force field tended to produce a more stable structure. The PEG-4 crowding had little effect on the Coulomb contribution to the intramolecular energies of the protein, while the vdW contribution appeared to increase with the PEG-4 concentration (less favorable). Because the vdW part contributed little, the total intramolecular interactions of the protein seemed insensitive to the crowder concentration, except a slight decrease for the case of a99SB-*disp* simulations at 370 K (Figure S4).

#### 2.3.5. Solvent Accessible Surface Area

**Figure 10:**
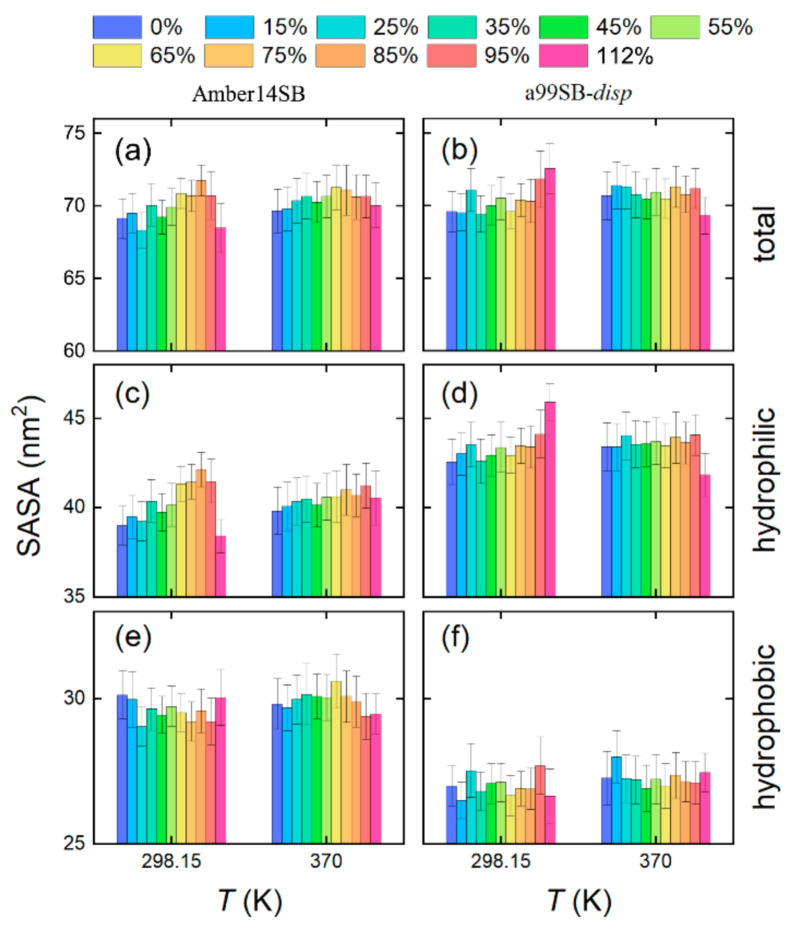
Solvent accessible surface area (SASA) of lysozyme in different concentrations of PEG-4 solutions using Amber14SB (**left**) and a99SB-*disp* (**right**) force fields at *T* = 298.15 and 370 K. The total SASAs (**a**,**b**) are decomposed into hydrophilic (**c**,**d**) and hydrophobic (**e**,**f**) parts. Protein atoms with an absolute charge smaller than 0.2 *e* are used for computing the hydrophobic SASA, while the others are for the hydrophilic SASA.

The total solvent accessible surface area (SASA) of the lysozyme appeared insensitive to the PEG-4 crowding, and no obvious changes in the SASA were observed in different PEG-4 concentrations, as shown in panels a and b of Figure 10 for the Amber14SB and a99SB-*disp* force fields, respectively. The a99SB-*disp* force field allowed more hydrophilic contacts between lysozyme and solvent molecules than the Amber14SB, as indicated by the hydrophilic SASA (Figure 10, panels c and d). On the contrary, the Amber14SB tended to favor hydrophobic contacts between the protein and solvent molecules (Figure 10, panels e and f). These findings agreed with the observations of protein–solvent interactions in Figure 9 and Figure S3 and indicated that the PEG-4/a99SB-*disp* solutions were more hydrophilic than the PEG-4/TIP3P solutions. The crowder PEG-4 acted as a cosolvent and may offer both hydrophilic and hydrophobic interactions with lysozyme. Interestingly, the replacement of water by PEG-4 almost did not change the protein–solvent hydrophobic contacts, while the hydrophilic contacts appeared to increase slightly with the increasing PEG-4 concentration (Figure 10).

### 2.4. Crowding Effects on the Diffusivity of Protein Systems

**Figure 11:**
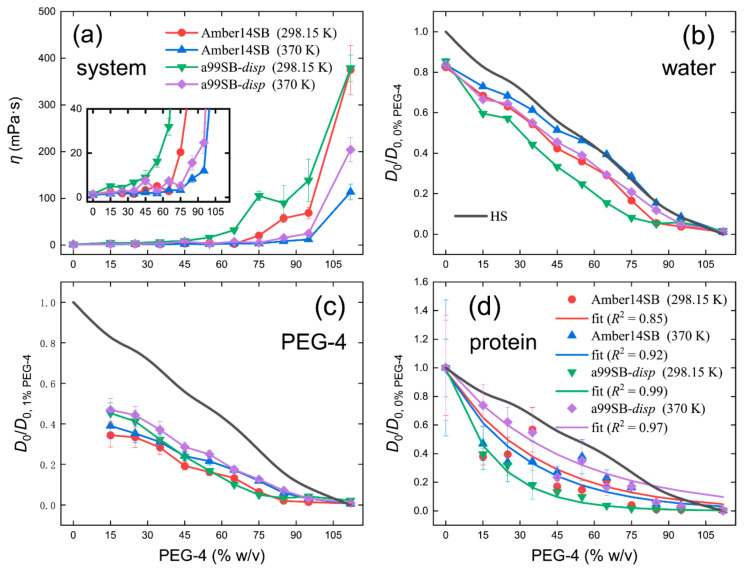
Viscosity of the simulated protein solution systems (**a**) and the diffusivity of water (**b**), PEG-4 (**c**), and protein (**c**) in different PEG-4 concentrations. The insert is an enlarged view for the viscosity (**a**). Diffusion constants in the infinite solution (*D*_0_) are normalized to be one in the cases of 0% and 1% *w/v* PEG-4 solutions for water (**a**) and PEG-4 (**b**), respectively. We used the case of 0% *w/v* protein/PEG-4 solutions for the normalization of protein constants (**c**). Black lines (**b**,**d**) indicate a decrease in the diffusion constants of the hard spheres (HS) predicted by the Enskog theory (Equation (1)). Exponential fits of simulated data points are given in panel (**d**), indicative of the decline in protein diffusivity. The calculated diffusion constants of water, PEG-4, and protein are given in Table S7, Table S8 and Table S9, respectively.

With the increasing PEG-4 concentration, the viscosities of protein/PEG-4/water systems increased from one to several hundred (Figure 11a and Table S5). Due to the presence of the protein, the system viscosities were largely reduced compared to the PEG-4 solutions (Table S1). The a99SB-*disp* force field still gave a large viscosity of protein systems due to the large viscosity of the a99SB-*disp* water model (Figure 11a). The system of 35% (*w/v*) was roughly equal to the physiological water concentration with a mass fraction of ca. 70% (Table S3). At this concentration, the system viscosity at 298.15 K increased with a factor of 4.7 and 7.6 relative to the pure water for the Amber14SB and a99SB-*disp* force fields, respectively; the latter agreed well with the in vivo nuclear magnetic resonance prediction with a factor of ca. 8 [86].

In the presence of lysozyme without the crowder PEG-4 (0% *w/v*), the water diffusivity was reduced to 84% of the pure water case on average (Figure 11b). At the physiological water concentration, water diffusivity was reduced by about 50% (Figure 11b), close to the prediction of ~40% in our previous work on the self-crowding of proteins [40]. Similar to the PEG-4 solutions (Figure 2), the water diffusivity predicted by the a99SB-*disp* force field dropped at a faster pace than the Amber14SB, likely due to the larger viscosity of the former than the latter. The decrease in the water diffusivity slightly underestimated the prediction of hard spheres by the Enskog theory [80,81]. A large influence on the PEG-4 diffusivity was observed; at the physiological water concentration, the diffusion of PEG-4 was reduced by 70% relative to the dilute concentration of 1% *w/v*, as shown in Figure 11c. The hard sphere model largely overestimated the decline in the PEG-4 diffusivity in crowded environments.

Protein diffusivity was slowed down as well in the solutions crowed by PEG-4, and the decline in the diffusion appeared to follow an exponential trend with the correlation coefficients of *R*^2^ ≥ 0.85 (Figure 11d). The slow-down of protein diffusion was slightly less than the prediction by a hard sphere model that neglected attractive interactions. At the physiological water concentration, the protein diffusions at 298.15 K were reduced to 57% and 18% of the dilute values for the simulations with Amber14SB and a99SB-*disp* force fields, respectively. Based on the observation that the diffusion constant of green fluorescent protein in the cytoplasm was 7% of the in vitro value [87], the prediction with the a99SB-*disp* force field might be much closer to the in vivo measurement.

## 3. Computational Methods

### 3.1. Simulation Protocol

Molecular dynamics (MD) simulations were performed with GROMACS software [88]. Periodic boundary conditions in all directions were considered in the simulations. The particle-mesh Ewald (PME) method [89,90] was used to handle the Coulomb interactions. Nonbonded interactions were truncated at 1.0 nm, and a long-range dispersion correction for energy and pressure was applied for van der Waals (vdW) interactions. The Parrinello–Rahman algorithm [91,92] was used to couple the pressure at 1 bar with a coupling time constant of 5 ps, and the velocity-rescaling [93] was used to maintain the temperature at 298.15 K and 370 K with a coupling constant of 1 ps. The LINCS algorithm was applied to constrain all bonds [94], allowing a time step of 2 fs. Energy minimization was carried out to avoid the possible bad contacts in the initial configuration, followed by 100 ps NVT and 400 ps NPT. The production simulations were then implemented at NPT, and the trajectories were saved to disk every 500 steps (1 ps) for data analysis.

### 3.2. PEG-4 Solutions

We extracted the molecular structure of tetraethylene glycol (PEG-4) from the PubChem database [95] and then optimized it at the level of HF/6-31G* in gas phase with the Gaussian 09 software [96]. The Gaussian output file was imported into the “antechamber” tool [97] for computing the restrained electrostatic potential (RESP) charges. The general Amber force field (GAFF) [98] was used to model the crowder PEG-4. The concentrations of PEG-4 solutions ranged from 0% to 112% *w/v*, and each concentration was simulated for 10 ns in a cubic box with an image distance of 4 nm. The final coordinates were used as a solvent box for the simulation of lysozyme. The components of simulated PEG-4 solutions are listed in Table 1, where other units of concentrations (v% and wt%) are given as well. The unit of *w/v* is used in this work, and a concentration of 112% *w/v* corresponds to a pure liquid of PEG-4.

For the calculation of shear viscosity of PEG-4 solutions, we used non-equilibrium simulations via adding an acceleration $a_{x}\left(z\right)$ in the *x*-direction as a function of the *z*-coordinate (Equation (2)).

(2) $$a_{x}\left(z\right)\;=\;A\cos\left(\frac{2\pi z}{l_{z}}\right)$$

where *A* is the amplitude of the acceleration profile and was set to 0.05 nm ps^−2^, and *l*_z_ is the height of the simulation box. The adding small force leads to a velocity gradient, and the resulting velocity profile (*v*) is related to the viscosity (*η*) via Equation (3) [99,100,101].

(3) $$\eta =\rho \frac{a}{v}{\left(\frac{l_{z}}{2\pi }\right)}^{2}$$

where *ρ* is the solution density. These non-equilibrium simulations were extended to 50 ns for the *η* convergence. Note that the non-equilibrium simulations can only be used to compute one property, as done in this work for the viscosity.

**Table 1:** System components of PEG-4 solutions in different concentrations.

Concentration of PEG-4 Solutions			*N*_PEG-4_	*N*_water_
% *w/v*	v%	wt%		
0	0	0	0	2139
15	13	14	29	1859
25	22	24	49	1666
35	31	33	69	1474
45	40	42	89	1281
55	49	51	109	1088
65	57	60	128	905
75	66	69	148	713
85	75	77	168	520
95	84	86	188	327
112	100	100	222	0

The simulation box was 4 × 4 × 4 nm^3^, and a density of 1.12 and 1 g/mL for PEG-4 and water was used to compute the required number (N) of solvent molecules, respectively.

The 10 ns equilibrium production simulations at NPT were used to calculate the density (*ρ*) of PEG-4 solutions and the diffusion constants of solvent molecules. The diffusion constants (*D*_PBC_) of PEG-4 and water under periodic boundary conditions (PBC) were calculated from their mean squared displacements through the Einstein formula (Equation (4)) [102].

(4) $$D_{P\mathrm{BC}}=\underset{t\to \infty }{\lim}\frac{1}{6t}\langle \|r_{i}\left(t\right)\;-\;r_{i}\left(0\right)∥\rangle$$

where *t* is the simulation time and ***r*** is the position vector of the components. Box size-dependent finite-size effects needed to be corrected to obtain a prediction at infinite solutions (*D*_0_) [103,104]. *D*_0_ of PEG-4 and water molecules was computed by Equation (5).

(5) $$D_{0}=D_{\mathrm{PBC}}+\frac{k_{B}T\xi }{6\pi \eta L}$$

where *k*_B_ is the Boltzmann’s constant, *T* is the absolute temperature, ξ is a constant of 2.837297, *η* is the solution viscosity by Equation (3), and *L* is the length of the simulation box. We also performed MD simulations of one PEG-4 molecule in 2129 water molecules (a concentration of 1% *w/v*) and obtained the diffusion constants of PEG-4 for comparison with that in crowded environments.

### 3.3. Protein Systems

Crystal structure of hen egg white lysozyme (PDB code: 1AKI) with a resolution of 1.50 Å and a sequence length of 129 amino acids was used as an initial coordinate for protein simulations. The protein and the 78 crystal water molecules were placed in a cubic box with a length of 6 nm. For pure water simulations (0% *w/v*), the simulated box contains one protein, 6356 water molecules, and 8 Cl^−^ ions for neutrality. For the concentration of 112% *w/v*, the box was filled with one protein, 449 PEG-4 molecules, 70 water molecules, and 8 Cl^−^ ions. Two Amber-like force fields of Amber14SB [74] and a99SB-*disp* [66] were chosen to model the protein, in conjunction with TIP3P [69] and a99SB-*disp* [66] water models, respectively. A rigid water model was used with the SETTLE constraint [105]. In the NVT and NPT equilibrium stages, a harmonic potential with a force constant of 1000 kJ mol^−1^ nm^−2^ was exerted on the protein backbone atoms for position constraints, allowing equilibration of solvent molecules. Production simulations were run for 150 ns at NPT, and the last 50 ns were used for structural and thermodynamic analysis.

The shear viscosity of the protein systems was computed via the Einstein relation [40,99] from the equilibrium simulations of lysozyme in PEG-4 solutions (Equation (6)).

(6) $$\eta =\frac{1}{2}\frac{V}{k_{B}T}\underset{t\to \infty }{\lim}\frac{d}{dt}\frac{1}{6}\sum_{i=1}^{3}\sum_{j=1,i\neq j}^{3}{\langle \left(\int_{t_{0}}^{t_{0}+t}P_{ij}\left(t^{\prime }\right)dt^{\prime }\right)}^{2}\rangle_{t_{0}}$$

where *V* is the system volume and *P*_ij_ indicates the six off-diagonal pressure tensor components. This was computed via the GROMACS utility of “gmx energy” [88]. With the system viscosity, Equation (5) was used to compute the diffusion constants (*D*_0_) of PEG-4 and water in the presence of lysozyme. For the *D*_0_ of proteins, an additional correction for the dependence on the particle size was needed (Equation (7)).

(7) $$D_{0}=D_{\mathrm{PBC}}+\frac{k_{B}T}{6\pi \eta L}\left(\xi -\frac{4\pi R^{2}}{3L^{2}}\right)$$

where *R* is the hydrodynamic radius of lysozyme and we used the experimental estimation of 1.87 nm [106]. Errors of viscosity and diffusion results were calculated with block averaging by dividing the last 100 ns trajectories into five blocks.

## 4. Concluding Remarks

Here we used tetraethylene glycol (PEG-4) as a small molecule crowder and simulated a model protein of lysozyme at a wide concentration of PEG-4 solutions from diluted water to a pure PEG-4 liquid using two Amber-like force fields of Amber14SB [74] and a99SB-*disp* [66]. The former force field was often used with its native water model of TIP3P [69], although it was argued to fail in reproducing the kinetic properties of interest due to the known drawbacks of fast diffusivity and low viscosity. The latter one was designed for both folded and disordered proteins and used the a99SB-*disp* water model [66]. As inspired by the TIP4P-D model [63], this model used a large dispersion coefficient and was expected to strengthen protein–water interactions. Increasing the simulated temperature to 370 K, we still did not observe obvious changes in the lysozyme conformation using the Amber14SB force field. This was due to the force field model generating too weak protein–solvent interactions and too stable protein structures. For the a99SB-*disp* force field, surprisingly, the lysozyme structure was also maintained well in different PEG-4 concentrations at 298.15 K, probably due to the high viscosity of the used water model. Increasing the temperature reduced the system viscosity, which allowed the strong protein–water interactions and weak intramolecular interactions to affect the protein stability. Even so, the protein structure still remained intact at high PEG-4 concentrations because of the crowding effects and/or the increased viscosities.

The diffusion of protein and solvent molecules (PEG-4 + water) highly depended on the viscosity of simulated systems. The Amber14SB and a99SB-*disp* force fields appeared to overestimate the system viscosity largely, which indicated that our calculated diffusion constants deviated much from the reality (probably too small). We instead used the normalized diffusion to eliminate the possible viscosity errors and obtain a meaningful comparison at different concentrations. The observed slow-down in the diffusion constants of PEG-4, water, and protein from our atomistic simulations were more in most cases than the prediction by the hard sphere (HS) model due to the HS model neglecting attractive (electrostatic) interactions.

This work highlighted the importance of force fields in the modeling of molecular crowding. Compared to the Amber14SB force field, the a99SB-*disp* model produced more coordinated water and fewer PEG-4 molecules around lysozyme, less intramolecular hydrogen bonds (HBs) in the protein, more protein–water HBs, and less protein–PEG HBs, as well as stronger interactions and more hydrophilic and less hydrophobic contacts with solvent molecules. Our results supported the use of the a99SB-*disp* force field to describe protein diffusion in a crowding environment. The latest Amber force field ff19SB was recommended to use with the OPC water model (also having a large dispersion coefficient) [64] for improved performance in the simulation of discorded proteins [65]. This force field/water combination is likely applicable to the crowding conditions as well. Here we chose a small molecule crowder and a small model protein to investigate the crowding effects on the structural and diffusion properties. For large crowder-like proteins, more copies of the crowders are needed, occupying at least the first solvation shell of the biomolecules under study, and modeling such cases in atomistic detail is computationally demanding. Instead, simplified models, such as the single-particle or many-particle crowders, allow large-scale crowding simulations, although the models were argued to be oversimplified and insufficient to capture the specific nature of the interactions with the crowders [23].

## Data Availability

Data is contained within the article.

## Supplementary Materials

The following supporting information can be downloaded at: https://www.mdpi.com/article/10.3390/molecules27072110/s1, Tables S1 and S2: viscosity and water diffusion in PEG-4 solutions; Table S3: volume and mass fraction of the simulated systems; Table S4: PEG-4 diffusion in PEG-4 solutions; Table S5: viscosity of protein/PEG-4 solutions; Table S6: calculated proportion of protein secondary structures; Tables S7–S9: the diffusion constants of water, PEG-4, and lysozyme in protein/PEG-4 solutions; Figure S1: the convergence of viscosity calculation; Figure S2: distribution of protein RMSDs; Figure S3: interaction energies between protein with solvent molecules; Figure S4: intramolecular interaction energies of lysozyme.

## Sample Availability

Force field parameters of the compound PEG-4 are available from the authors.
