# Supplementary material for: Atomistic Simulation of Lysozyme in Solutions Crowded by Tetraethylene Glycol: Force Field Dependence
Source: Molecules. 2022 Mar 25;27(7):2110. doi: 10.3390/molecules27072110 (PMC9000840; doi:10.3390/molecules27072110)
Supplement: Supplementary file 1 [file molecules-27-02110-s001.zip › molecules-1631234-supplementary.pdf]

Supporting Information for

Atomistic Simulation of Lysozyme in Solutions Crowded by Tetraethylene Glycol: Force Field Dependence

*Donglin Liu, Yejie Qiu, Qing Li, and Haiyang Zhang**

Department of Biological Science and Engineering, School of Chemistry and Biological Engineering, University of Science and Technology Beijing, 100083 Beijing, China

Corresponding Author

*zhanghy@ustb.edu.cn

Table S1. Calculated Viscosities (mPa•s) of PEG-4 Solutions with Different Concentrations[a].

concentration	Amber14SB		a99SB-*disp*		exp[b]
(% w/v)	T = 298.15 K	T = 370 K	T = 298.15 K	T = 370 K	T = 298.15 K
0	0.31 ± 0.01	0.16 ± 0.01	1.01 ± 0.01	0.36 ± 0.01	0.89
15	0.49 ± 0.01	0.23 ± 0.01	1.81 ± 0.01	0.54 ± 0.01	1.56
25	0.72 ± 0.01	0.31 ± 0.01	2.92 ± 0.01	0.75 ± 0.01	2.30
35	1.14 ± 0.01	0.42 ± 0.01	5.31 ± 0.02	1.08 ± 0.01	3.42
45	2.07 ± 0.01	0.61 ± 0.01	11.36 ± 0.06	1.65 ± 0.01	4.99
55	4.31 ± 0.02	0.93 ± 0.01	29.99 ± 0.33	2.69 ± 0.01	7.21
65	10.98 ± 0.03	1.50 ± 0.01	95.68 ± 0.65	4.63 ± 0.01	10.36
75	37.98 ± 0.11	2.68 ± 0.01	525.88 ± 14.42	8.61 ± 0.03	14.89
85	223.72 ± 4.44	5.66 ± 0.01	1871.10 ± 118.00	18.21 ± 0.20	22.50
95	1185.67 ± 62.33	13.74 ± 0.05	2654.89 ± 137.94	37.55 ± 0.27	31.80
112	4057.45 ± 279.95	109.42 ± 1.05	4057.45 ± 279.95	109.42 ± 1.05	44.63

[a]Model parameters of PEG-4 are identical for the both Amber-like force fields. The viscosities were computed by eq 3 from non-equilibrium simulations. The concentration of 112% w/v means a pure PEG-4 liquid. [b]Obtained from polynomial fits of experimental observations [1].

Table S2. Calculated Diffusion Constants (x 10^{-5} cm^2/s) of Water in PEG-4 Solutions with Different Concentrations[a].

concentration	Amber14SB		a99SB-*disp*	
(% w/v)	T = 298.15 K	T = 370 K	T = 298.15 K	T = 370 K
0	6.13 ± 0.10	13.80 ± 0.31	1.91 ± 0.08	6.37 ± 0.15
15	4.37 ± 0.11	10.95 ± 0.24	1.27 ± 0.04	4.77 ± 0.14
25	3.40 ± 0.10	8.97 ± 0.20	0.89 ± 0.04	3.83 ± 0.10
35	2.44 ± 0.07	7.25 ± 0.23	0.57 ± 0.03	2.92 ± 0.08
45	1.67 ± 0.08	5.71 ± 0.18	0.35 ± 0.02	2.18 ± 0.09
55	1.06 ± 0.06	4.29 ± 0.14	0.19 ± 0.02	1.54 ± 0.06
65	0.58 ± 0.04	3.11 ± 0.14	0.09 ± 0.01	1.05 ± 0.04
75	0.26 ± 0.02	2.00 ± 0.11	0.06 ± 0.01	0.63 ± 0.03
85	0.19 ± 0.04	1.17 ± 0.08	0.06 ± 0.01	0.36 ± 0.04
95	0.13 ± 0.02	0.62 ± 0.03	0.06 ± 0.01	0.21 ± 0.03
112	-	-	-	-

[a]Computed by eq 5 using the viscosity in Table S1. The concentration of 112% w/v means a pure PEG-4 liquid.

Table S3. Volume Fraction (%) of the Crowder PEG-4 and Mass Fraction (%) of Non-Water Components for the Simulated Systems of PEG-4 Solutions and of Protein/PEG-4 Solutions[a].

concentration	PEG-4 solutions		protein/PEG-4 solutions	
(% w/v)	volume	mass	volume	mass
0	0.0 ± 0.0	0.0 ± 0.0	0.0 ± 0.0	11.4 ± 0.1
15	12.4 ± 0.4	14.4 ± 0.0	7.9 ± 1.3	20.4 ± 1.2
25	21.1 ± 0.6	24.1 ± 0.0	9.7 ± 0.5	22.7 ± 0.3
35	30.1 ± 0.8	33.5 ± 0.0	14.8 ± 1.1	28.7 ± 0.8
45	39.2 ± 1.0	42.8 ± 0.0	21.0 ± 0.7	35.5 ± 0.2
55	48.5 ± 1.3	51.9 ± 0.0	25.3 ± 1.4	40.3 ± 0.8
65	57.5 ± 1.5	60.4 ± 0.0	31.0 ± 2.5	46.9 ± 1.8
75	66.9 ± 1.4	69.1 ± 0.0	39.6 ± 1.5	55.7 ± 1.0
85	76.1 ± 1.2	77.7 ± 0.0	51.9 ± 1.1	67.4 ± 0.7
95	84.9 ± 0.6	86.1 ± 0.0	62.7 ± 1.0	78.3 ± 0.7
112	100.0 ± 0.0	100.0 ± 0.0	86.1 ± 0.5	98.8 ± 0.0

[a]Fractions were averaged over the four simulation systems (two force fields and two temperatures). Standard deviations of 0.0 indicate a value of < 0.1. The volume factions were used to estimate a decrease in the diffusion coefficient for hard spheres based on the Enskog theory.

Table S4. Calculated Diffusion Constants (x 10^{-5} cm^2/s) of PEG-4 in PEG-4 Solutions with Different Concentrations[a].

concentration	Amber14SB		a99SB-*disp*	
(% w/v)	T = 298.15 K	T = 370 K	T = 298.15 K	T = 370 K
0	-	-	-	-
1	2.05 ± 0.75	5.36 ± 1.91	0.60 ± 0.24	2.16 ± 0.74
15	1.22 ± 0.18	3.34 ± 0.38	0.44 ± 0.07	1.49 ± 0.08
25	0.89 ± 0.08	2.41 ± 0.32	0.28 ± 0.03	1.14 ± 0.13
35	0.61 ± 0.05	1.85 ± 0.20	0.18 ± 0.02	0.84 ± 0.07
45	0.40 ± 0.06	1.41 ± 0.10	0.10 ± 0.01	0.65 ± 0.07
55	0.21 ± 0.02	0.98 ± 0.08	0.05 ± 0.01	0.46 ± 0.03
65	0.11 ± 0.01	0.70 ± 0.04	0.02 ± 0.01	0.32 ± 0.02
75	0.04 ± 0.01	0.45 ± 0.03	0.02 ± 0.01	0.19 ± 0.02
85	0.03 ± 0.01	0.27 ± 0.02	0.02 ± 0.01	0.11 ± 0.01
95	0.02 ± 0.01	0.15 ± 0.01	0.02 ± 0.01	0.07 ± 0.01
112	0.01 ± 0.01	0.04 ± 0.01	0.01 ± 0.01	0.04 ± 0.01

[a]Computed by eq 5 using the viscosity in Table S1. The concentration of 112% w/v means a pure PEG-4 liquid. The system of 1% w/v contains one PEG-4 molecule and 2129 water molecules.

Table S5. Calculated Viscosities (mPa•s) of Protein/PEG-4 Solutions with Different Concentrations[a].

concentration	Amber14SB		a99SB-*disp*	
(% w/v)	T = 298.15 K	T = 370 K	T = 298.15 K	T = 370 K
0	1.36 ± 0.23	1.04 ± 0.07	1.57 ± 0.14	1.50 ± 0.12
15	2.60 ± 0.17	1.58 ± 0.11	5.10 ± 0.92	2.09 ± 0.18
25	1.85 ± 0.25	2.40 ± 0.17	4.47 ± 0.75	2.85 ± 0.46
35	1.47 ± 0.24	1.58 ± 0.13	6.76 ± 0.89	2.90 ± 0.30
45	3.37 ± 0.91	2.22 ± 0.69	9.00 ± 3.19	7.61 ± 1.28
55	5.03 ± 0.70	1.73 ± 0.17	16.26 ± 2.62	2.89 ± 0.37
65	2.77 ± 0.66	3.15 ± 0.25	31.97 ± 3.86	7.37 ± 0.87
75	20.46 ± 1.26	3.26 ± 0.72	105.27 ± 10.03	5.10 ± 1.85
85	57.42 ± 9.19	8.39 ± 1.63	89.82 ± 37.86	15.63 ± 3.24
95	68.75 ± 12.17	11.98 ± 0.99	138.76 ± 45.21	24.70 ± 4.81
112	374.87 ± 52.93	113.57 ± 16.89	378.62 ± 28.43	204.39 ± 26.03

[a]The viscosities were computed by eq 6 from equilibrium simulations. There are 70 water moelcules in the 112% w/v solution.

Table S6. Comparison of Simulated Proportion of Lysozyme Secondary Structures with the Crystal Structure.

PEG (% w/v)	α-helix (%)		β-sheet (%)		β-bridge (%)		turn (%)		total (%)	
	298.15 K	370 K	298.15 K	370 K	298.15 K	370 K	298.15	370 K	298.15	370 K
					Amber14SB					
0	30.7 ± 0.7	31.2 ± 0.2	6.3 ± 0.1	6.8 ± 0.0	4.3 ± 0.0	3.9 ± 0.0	25.4 ± 0.3	25.0 ± 0.4	66.7 ± 0.6	66.8 ± 0.2
15	31.9 ± 0.3	31.0 ± 0.2	6.7 ± 0.0	6.8 ± 0.0	4.1 ± 0.0	3.9 ± 0.0	26.1 ± 0.8	24.9 ± 0.2	68.8 ± 0.8	66.6 ± 0.2
25	33.2 ± 0.1	30.9 ± 0.2	6.8 ± 0.1	6.7 ± 0.0	4.1 ± 0.0	3.8 ± 0.1	24.6 ± 0.1	25.3 ± 0.2	68.6 ± 0.1	66.7 ± 0.2
35	31.8 ± 0.4	32.0 ± 0.2	7.1 ± 0.1	6.9 ± 0.0	4.0 ± 0.1	3.7 ± 0.2	24.8 ± 0.6	23.6 ± 0.2	67.6 ± 0.2	66.1 ± 0.2
45	33.7 ± 0.1	30.9 ± 0.3	6.8 ± 0.1	7.0 ± 0.1	4.1 ± 0.1	3.0 ± 0.1	24.2 ± 0.3	24.6 ± 1.0	68.8 ± 0.4	65.4 ± 0.6
55	30.9 ± 0.2	31.2 ± 0.1	7.1 ± 0.1	7.0 ± 0.1	4.0 ± 0.1	3.8 ± 0.1	25.8 ± 0.1	24.0 ± 0.8	67.7 ± 0.2	65.9 ± 0.8
65	30.1 ± 0.1	29.5 ± 0.7	6.7 ± 0.0	6.5 ± 0.1	3.1 ± 0.0	4.0 ± 0.1	25.0 ± 0.2	25.8 ± 0.3	64.9 ± 0.2	65.8 ± 0.9
75	31.1 ± 0.1	30.7 ± 0.4	6.8 ± 0.1	6.8 ± 0.1	4.0 ± 0.1	3.9 ± 0.0	26.2 ± 0.1	25.8 ± 0.3	68.1 ± 0.2	67.2 ± 0.3
85	31.2 ± 0.4	31.4 ± 0.3	7.1 ± 0.5	6.9 ± 0.1	3.9 ± 0.1	3.6 ± 0.3	25.6 ± 0.2	25.0 ± 1.5	67.9 ± 0.5	66.9 ± 1.4
95	31.3 ± 0.6	31.3 ± 0.1	7.0 ± 0.2	7.1 ± 0.0	3.7 ± 0.1	3.8 ± 0.0	25.5 ± 0.2	25.0 ± 0.2	67.6 ± 1.4	67.1 ± 0.2
112	30.0 ± 0.2	31.2 ± 0.6	6.5 ± 0.1	6.8 ± 0.1	3.9 ± 0.1	3.8 ± 0.1	25.5 ± 0.3	25.8 ± 0.8	65.8 ± 0.2	67.6 ± 0.2
					a99SB-*disp*					
0	29.9 ± 0.1	29.7 ± 0.3	6.8 ± 0.1	6.8 ± 0.1	4.0 ± 0.1	3.2 ± 0.1	25.4 ± 0.1	25.4 ± 0.5	66.2 ± 0.1	65.1 ± 0.9
15	32.0 ± 0.2	29.2 ± 0.2	7.0 ± 0.1	6.6 ± 0.1	4.0 ± 0.0	4.0 ± 0.1	23.9 ± 0.3	24.0 ± 0.1	66.9 ± 0.4	63.7 ± 0.2
25	30.5 ± 0.2	30.9 ± 0.3	6.8 ± 0.1	6.9 ± 0.1	4.0 ± 0.0	3.9 ± 0.1	25.0 ± 0.1	24.0 ± 0.5	66.3 ± 0.2	65.6 ± 0.3
35	32.1 ± 0.1	29.2 ± 0.2	7.2 ± 0.2	6.8 ± 0.0	3.9 ± 0.1	3.7 ± 0.1	24.6 ± 0.3	24.0 ± 0.3	67.8 ± 0.3	63.8 ± 0.6
45	33.1 ± 0.6	31.5 ± 0.3	7.1 ± 0.1	6.9 ± 0.0	3.9 ± 0.0	3.9 ± 0.0	23.4 ± 0.2	24.4 ± 0.3	67.5 ± 0.8	66.7 ± 0.2
55	30.5 ± 0.8	31.2 ± 0.1	6.8 ± 0.0	6.6 ± 0.0	4.1 ± 0.0	4.1 ± 0.0	24.4 ± 0.2	23.8 ± 0.2	65.8 ± 0.7	65.6 ± 0.5
65	31.3 ± 0.2	31.1 ± 0.1	7.0 ± 0.0	6.9 ± 0.0	4.0 ± 0.0	3.8 ± 0.0	24.3 ± 0.2	24.9 ± 0.1	66.6 ± 0.3	66.8 ± 0.1
75	31.9 ± 0.1	31.2 ± 0.1	7.7 ± 0.2	6.8 ± 0.1	3.8 ± 0.1	3.1 ± 0.0	25.4 ± 0.2	22.9 ± 0.2	68.8 ± 0.3	64.1 ± 0.4
85	29.2 ± 0.3	31.5 ± 0.1	6.6 ± 0.0	7.0 ± 0.0	4.1 ± 0.1	3.9 ± 0.0	26.5 ± 0.5	24.1 ± 0.2	66.4 ± 0.2	66.5 ± 0.2
95	30.1 ± 0.2	29.9 ± 0.1	7.4 ± 0.1	6.8 ± 0.1	3.8 ± 0.1	3.9 ± 0.0	23.9 ± 0.6	26.0 ± 0.5	65.2 ± 0.8	66.6 ± 0.4
112	31.2 ± 0.2	31.2 ± 0.2	7.4 ± 0.2	6.7 ± 0.1	3.7 ± 0.1	3.9 ± 0.1	26.0 ± 0.2	25.6 ± 0.3	68.2 ± 0.3	67.3 ± 0.4
crystal	30.2		6.2		4.7		26.4		67.4	

Table S7. Calculated Diffusion Constants (x 10^{-5} cm^2/s) of water in Protein/PEG-4 Solutions with Different Concentrations[a].

concentration	Amber14SB		a99SB-*disp*	
(% w/v)	*T* = 298.15 K	*T* = 370 K	*T* = 298.15 K	*T* = 370 K
0	5.05 ± 0.07	11.55 ± 0.16	1.63 ± 0.03	5.33 ± 0.08
15	4.19 ± 0.06	10.06 ± 0.15	1.14 ± 0.02	4.25 ± 0.06
25	3.87 ± 0.05	9.43 ± 0.15	1.09 ± 0.02	4.11 ± 0.06
35	3.34 ± 0.06	8.45 ± 0.13	0.85 ± 0.02	3.50 ± 0.05
45	2.59 ± 0.05	7.09 ± 0.11	0.64 ± 0.01	2.90 ± 0.04
55	2.20 ± 0.05	6.39 ± 0.10	0.47 ± 0.01	2.48 ± 0.04
65	1.79 ± 0.04	5.44 ± 0.12	0.29 ± 0.01	1.86 ± 0.04
75	1.02 ± 0.03	3.93 ± 0.08	0.15 ± 0.01	1.32 ± 0.03
85	0.34 ± 0.01	2.13 ± 0.06	0.10 ± 0.01	0.75 ± 0.02
95	0.22 ± 0.01	1.17 ± 0.04	0.11 ± 0.01	0.31 ± 0.01
112	0.07 ± 0.02	0.14 ± 0.02	0.03 ± 0.01	0.09 ± 0.01

[a] Computed by eq 5 using the viscosity in Table S4. There are 70 water molecules in the 112% w/v solution.

Table S8. Calculated Diffusion Constants (x 10^{-5} cm^2/s) of PEG-4 in Protein/PEG-4 Solutions with Different Concentrations.

concentration	Amber14SB		a99SB-*disp*	
(% w/v)	*T* = 298.15 K	*T* = 370 K	*T* = 298.15 K	*T* = 370 K
0	-	-	-	-
15	0.70 ± 0.12	2.09 ± 0.33	0.27 ± 0.03	1.01 ± 0.12
25	0.68 ± 0.10	1.89 ± 0.24	0.25 ± 0.03	0.96 ± 0.09
35	0.58 ± 0.07	1.66 ± 0.18	0.19 ± 0.02	0.80 ± 0.09
45	0.40 ± 0.04	1.29 ± 0.12	0.14 ± 0.01	0.62 ± 0.05
55	0.33 ± 0.04	1.16 ± 0.11	0.10 ± 0.01	0.54 ± 0.04
65	0.27 ± 0.02	0.93 ± 0.09	0.06 ± 0.01	0.38 ± 0.03
75	0.13 ± 0.01	0.64 ± 0.05	0.03 ± 0.01	0.27 ± 0.02
85	0.04 ± 0.01	0.32 ± 0.02	0.02 ± 0.01	0.15 ± 0.01
95	0.03 ± 0.01	0.17 ± 0.01	0.03 ± 0.01	0.07 ± 0.01
112	0.01 ± 0.01	0.03 ± 0.01	0.01 ± 0.01	0.02 ± 0.01

[a]Computed by eq 5 using the viscosity in Table S4. There are 70 water molecules in the 112% w/v solution.

Table S9. Calculated Diffusion Constants (x 10^{-7} cm^2/s) of Lysozyme in Protein/PEG-4 Solutions with Different Concentrations[a].

concentration	Amber14SB		a99SB-*disp*	
(% w/v)	T = 298.15 K	T = 370 K	T = 298.15 K	T = 370 K
0	23.08 ± 7.68	40.81 ± 19.41	12.01 ± 2.41	21.12 ± 7.76
15	8.76 ± 1.34	19.16 ± 4.87	4.76 ± 1.26	15.59 ± 3.01
25	9.12 ± 2.67	13.91 ± 5.69	3.84 ± 0.82	13.11 ± 2.19
35	13.12 ± 3.60	14.05 ± 5.91	2.19 ± 1.19	11.57 ± 3.65
45	3.95 ± 0.95	11.01 ± 1.72	1.59 ± 0.38	4.95 ± 1.17
55	3.42 ± 0.80	15.46 ± 4.90	1.17 ± 0.50	7.39 ± 2.94
65	4.90 ± 0.73	9.43 ± 2.27	0.46 ± 0.13	3.52 ± 1.23
75	0.94 ± 0.24	6.71 ± 1.41	0.20 ± 0.02	3.62 ± 0.65
85	0.26 ± 0.03	1.88 ± 0.30	0.17 ± 0.04	1.29 ± 0.21
95	0.19 ± 0.02	1.60 ± 0.55	0.13 ± 0.03	0.72 ± 0.10
112	0.05 ± 0.01	0.14 ± 0.01	0.04 ± 0.01	0.11 ± 0.02

[a]Computed by eq 7 using the viscosity in Table S4. There are 70 water molecules in the 112% w/v solution.

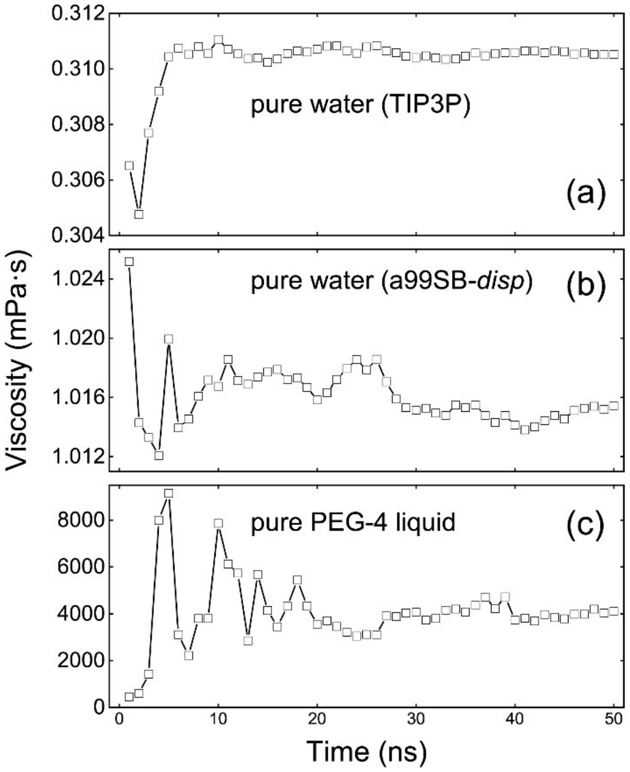

Figure S1. Calculated viscosities of the pure water using TIP3P (a) and a99SB-*disp* (b) models and pure PEG-4 liquid (c) as a function of simulation time.

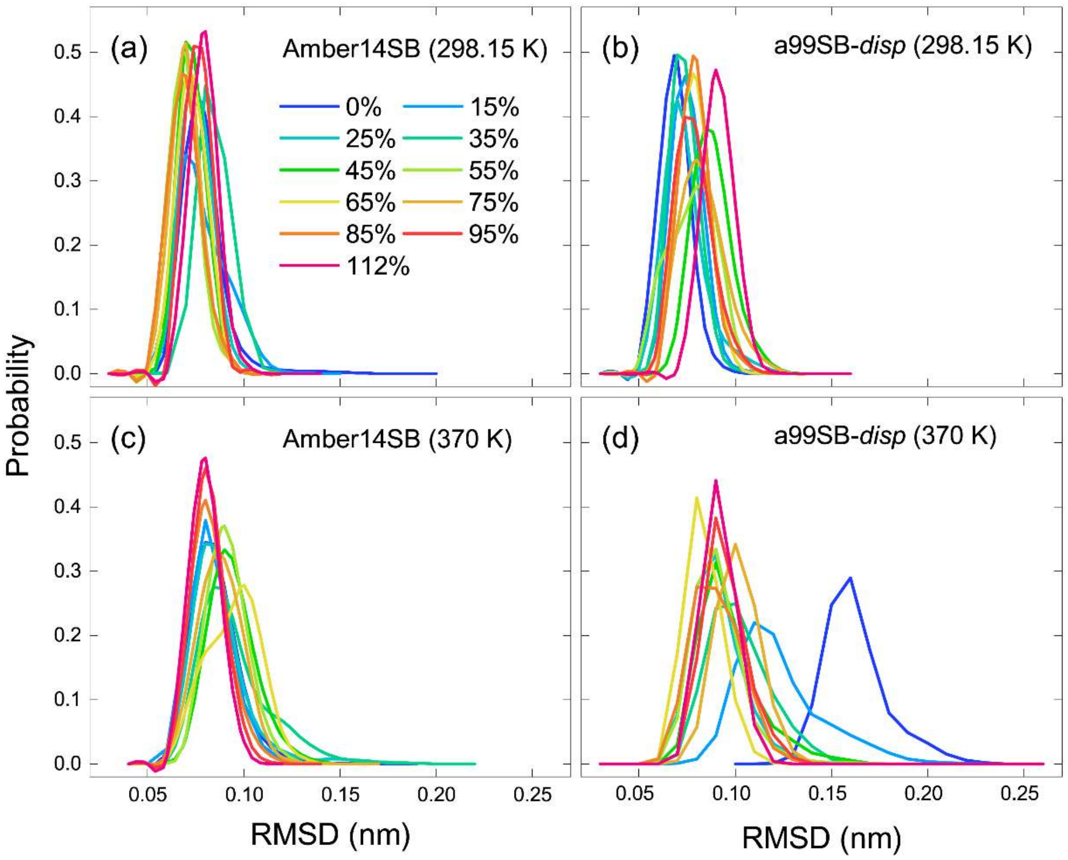

Figure S2. Probability distribution of the RMSDs for lysozyme using Amber14SB (*left*) and a99SB-*disp* (*right*) force fields during the simulations at 298.15 K (a-b) and 370 K (c-d).

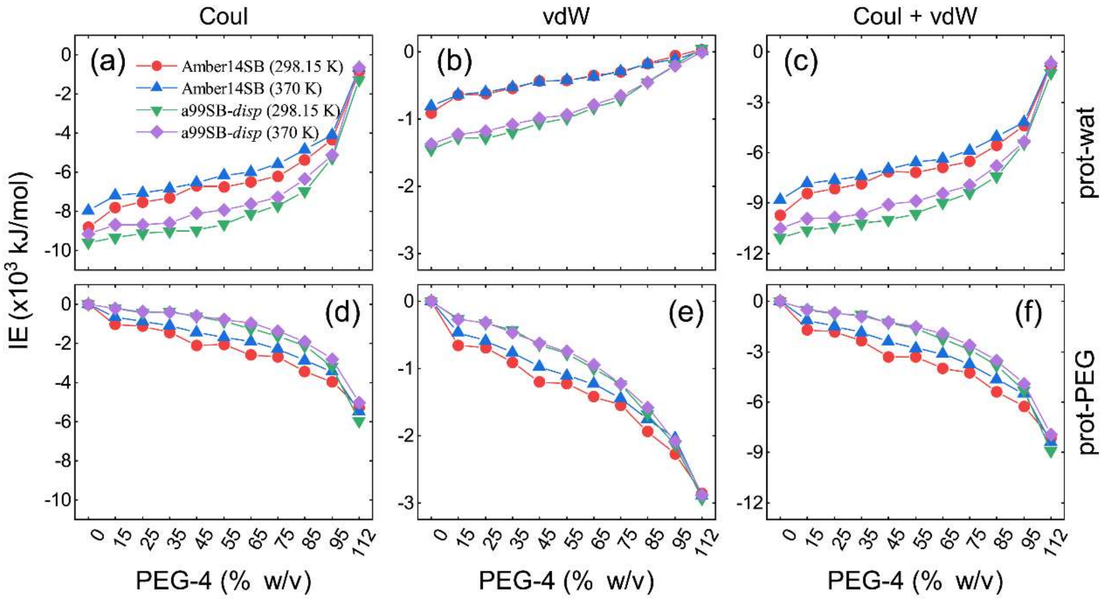

Figure S3. Interaction energy between lysozyme and solvent molecules of water (*top*) and PEG-4 (*bottom*) for the simulations of lysozyme in different PEG-4 concentrations using Amber14SB and a99SB-*disp* force fields at 298.15 K and 370 K.

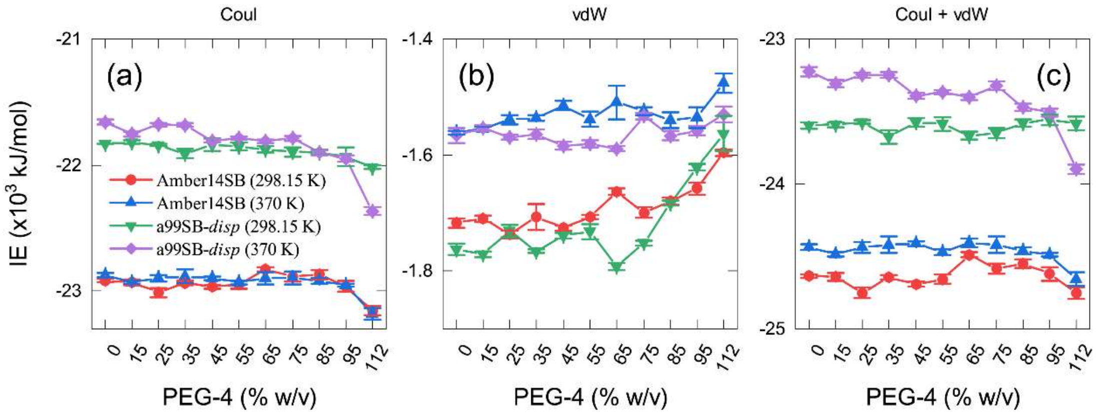

Figure S4. Intramolecular nonbonded interaction energy of protein for the simulations of lysozyme in different PEG-4 concentrations using Amber14SB and a99SB-*disp* force fields at 298.15 K and 370 K.

Reference

1. Liu, P.; Liu, Z.; Zhao, T.; Liu, F.; Liao, Q. Density, Viscosity, and Spectroscopic Nature for the Binary System of Tetraethylene Glycol (1) + Water (2) T = (298.15 to 323.15) K. *Int. J. Thermophys.* **2021**, *42*, 93. https://doi.org/10.1007/s10765-021-02851-8
